# YAP-activated NAT10 promotes hepatoblastoma progression by activating the pentose phosphate pathway

**DOI:** 10.7150/ijbs.109552

**Published:** 2025-04-13

**Authors:** Lingxiao Wang, Shiguang Yang, Jie Li, Yuan Fang, Mengzhou Guo, Xiaojing Du, Li Song, Sinuo Chen, Xingxing Zhang, Zhuoran Qi, Kaihui Zhang, Bei Lv, Jinglin Xia

**Affiliations:** 1 Department of General Pediatrics, Children's Hospital of Fudan University, National Children's Medical Center, Shanghai, China.; 2 National Medical Center & National Clinical Research Center for Interventional Medicine, Liver Cancer Institute, Zhongshan Hospital, Fudan University, Shanghai, China.; 3 Department of Hepatobiliary and Pancreatic Surgery, Minhang Hospital, Fudan University, Shanghai, China.; 4 Department of Radiation Oncology, Zhongshan Hospital, Fudan University, Shanghai, China.; 5 Institute of Pediatric Research, Children's Hospital Affiliated to Shandong University, Jinan, Shandong, China.; 6 Department of Liver Surgery, Key Laboratory of Carcinogenesis and Cancer Invasion (Ministry of Education), Liver Cancer Institute, Zhongshan Hospital,Fudan University, Shanghai, China.; 7 Department of Oncology, Zhongshan Hospital, Fudan University, Shanghai, China.; 8 Endoscopy Center, Shanghai East Hospital, Tongji University School of Medicine, Shanghai 200120, China.; 9 Department of Gastroenterology, Shanghai Jiaotong University Affiliated Sixth People Hospital South Campus, Shanghai, China.

**Keywords:** hepatoblastoma, N4-acetylcytidine, NAT10, pentose phosphate pathway, G6PD, YAP1

## Abstract

Hepatoblastoma (HB) is the most common malignant liver tumor in children, with limited treatment options. The N4-acetylcytidine (ac4C) modification, an important mRNA post-transcriptional modification catalyzed by N-acetyltransferase 10 (NAT10), plays a crucial role in the initiation and progression of tumors. However, its impact on the development and prognosis of HB is largely unknown. This study demonstrates that NAT10 is notably upregulated in HB. NAT10 inhibition suppressed HB proliferation and metastasis *in vitro* and *in vivo*. Mechanistically, Yes-associated protein 1 (YAP1) induced NAT10 transcription by binding to its promoter, which stimulates the ac4C modification within the 3' untranslated region (3' UTR) of glucose-6-phosphate dehydrogenase (G6PD) and enhancing its mRNA stability. YAP1/NAT10/G6PD axis resulted in enhanced pentose phosphate pathway (PPP) to promote proliferation and metastasis of HB. Moreover, said NAT10-mediated oncogenic effect could be significantly attenuated by a NAT10 inhibitor (Remodelin) both *in vitro* experiments and *in vivo* HB mouse models. Overall, our findings revealed the oncogenic role of NAT10 in regulating HB growth and metastasis, which can be a potential therapeutic target for human HB.

## Introduction

Hepatoblastoma (HB) represents the predominant pediatric hepatic malignancy, predominantly affecting children under four years of age and demonstrating aggressive biological behavior 1, 2. Currently, the main treatment strategies for hepatoblastoma involve multimodal therapies, primarily including surgery and chemotherapy 3. In certain cases, liver transplantation is combined with these treatments 4. Nevertheless, the prognosis for affected patients remains poor, characterized by high recurrence rates and an array of side effects 5. Therefore, there is an urgent need for the identification of novel biomarkers and therapeutic targets for the diagnosis and treatment of HB.

N4-acetylcytidine (ac4C) is a highly conserved chemical modification that is ubiquitously present in both eukaryotic and prokaryotic RNAs, including tRNA, rRNA, and mRNA 6, 7. This modification has been implicated in a variety of human diseases, particularly cancer 8, 9. N-acetyltransferase 10 (NAT10) is the first acetyltransferase which is responsible for ac4C mRNA modification, primarily located in the nucleus, and is crucial for cell growth, differentiation, and the stability and the translation efficiency of mRNA 10, 11. Previous studies have demonstrated that NAT10 expression is significantly increased in various cancer cells and tissues, correlating with the occurrence and progression of different malignancies 10, 12. For example, NAT10-mediated ac4C modification directly targets COL5A1, with NAT10 promoting gastric cancer metastasis 13. Additionally, in cervical cancer, NAT10-mediated ac4C modification upregulates the expression of FOXP1, thereby reprogramming the glycolytic metabolic pathway to promote malignant progression of cervical cancer 14. These findings suggest that NAT10-mediated ac4C modification plays a crucial part in cancer initiation and progression, but its specific mechanisms in hepatoblastoma remain unclear.

Metabolic reprogramming is a significant indicator of cancer progression, essential for cancer cell growth and energy acquisition 15. Pentose phosphate pathway (PPP), as a component of cellular metabolism, is a major pathway in glucose catabolism 16. PPP produce ribose for *de novo* nucleotide synthesis 17. And it also generates reduced forms of nicotinamide adenine dinucleotide phosphate (NADPH), which are utilized for lipid synthesis and reactive oxygen species (ROS) detoxification 17. Numerous studies indicate that PPP is abnormally activated in the progression of various cancers, particularly with significant overexpression of its key rate-limiting enzyme, Glucose-6-phosphate dehydrogenase (G6PD), promoting cancer cell growth and metastasis 16, 18, 19. However, the underlying mechanisms of ac4C in regulating PPP and biological function in HB are still poorly understood.

Yes-associated protein 1 (YAP1) is a transcriptional co-activator that acts as a key effector in the Hippo signaling pathway, playing an essential part in cell growth and organ development 20, 21. The studies have shown that YAP1 is overexpressed and activated in various cancers and tumor cells 22, 23. For example, YAP1 enhances the expression of SOX12 by upregulating FOXP4 in gastric cancer, thereby regulating cancer cell stemness and promoting tumor initiation and progression 22. Notably, YAP1 also plays a crucial part in reprogramming of glucose metabolism in breast cancer cells 24. However, the role of YAP1 in hepatoblastoma has been minimally studied, and its impact on metabolic reprogramming in hepatoblastoma cells requires in-depth research.

In this study, we demonstrated that NAT10 could promote the malignant progression of hepatoblastoma both *in vitro* and *in vivo*, mediating ac4C modification to upregulate G6PD expression and activate PPP. Additionally, NAT10 was primarily positively regulated by the upstream YAP1 signaling pathway and activated PPP, thereby influencing the metabolic reprogramming in hepatoblastoma, further promoting cancer cell proliferation, invasion, and metastasis. Furthermore, we discovered that the NAT10 inhibitor Remodelin effectively inhibited the malignant progression of hepatoblastoma. Overall, our findings indicates that NAT10 is a promising therapeutic target for conquering resistance in hepatoblastoma treatment.

## Materials and Methods

### Cell culture and treatment

HepG2, Huh6, HepT1, L-02 and other cell lines are derived from cell bank of Chinese Academy of Sciences. All cells were cultured in DMEM medium supplemented with 10% FBS. They were cultured in a incubator at 37 ºC with 5% CO_2_.Cells were cultured to 60%-80% confluence and then treated them with the Nuc, Nac, Verteporfin, Remodelin, and Fluasterone for drug treatments.

### Plasmids and cell transfection

The complete sequences of YAP1 were connected into the overexpression construct (pcDNA3.1). Sh-NAT10, Sh-G6PD, Sh-YAP1, and control sh-NC were compounded by GenePharma. Lipofectamine 3000 for cell transfection was used as the reagent. When the cell confluence reaches about 60%, mixing the plasmid with the transfection reagent and adding them to cells together, then continuing to culture them in an incubator at 37 ºC with 5% CO2.

### qRT-PCR assay

According to the instructions of the manufacturer, RNA from HB cells will be extracted using Trizol reagent (Ambion). Then, using a reverse transcription kit to convert the obtained RNA into cDNA samples. GAPDH was selected as the reference gene, and qRT-PCR amplification was performed by SYBR Green Mix (Genepharma), configured to obtain data. Finally, the relative expression of desired genes was figured out by using the 2-ΔΔCt calculation method.

### Western blot assay

The protein samples of HB cells were lysed by using RIPA lysis buffer and protease inhibitors on ice for 45 min, followed by centrifugation at 12,000 rpm for 12 min. The next, SDS was added for denaturation. Next, adding the protein marker and an equal amount of protein samples onto a 10% Tris-Bis gel, setting the working voltage to 110V. Afterward, using skim milk to block at room temperature for 2 h. Then, rabbit NAT10 (1:2000; Abcam), rabbit YAP1(1:1000; Abcam) and rabbit G6PD (1:1000; Abcam) were incubated at 4 ºC overnight. GAPDH was selected as the internal reference gene. Finally, chemiluminescence was performed and imaging was taken.

### Cell proliferation assays

5×10^3^ HB cells were seeded into a 96-well plate, and CCK-8 solution was added at different time points of cell culture, followed by incubation for 2 h. The absorbance was detected by using a microplate reader at 450 nm. HB cells were seeded into a 24-well plate and cultured in an incubator at 37 °C for 24 h with 5% CO2 before using an EDU detection kit to conduct the assay. For the colony formation assay, 1×10^3^ HB cells will be seeded into a 6-well plate. After incubation in an incubator at 37 °C for 14 d with 5% CO2, the cells were gently washed with PBS, then fixed with 4% paraformaldehyde for 25 min, the next, dyed with 0.5% crystal violet for 40 min, and finally, counting the number of colonies.

### Cell migration assays

For the Transwell assay, 1×10^6^ HB cells were seeded into the upper chamber of a 24-well polycarbonate Transwell. Then, 20% culture medium was placed in the lower chamber. After growing for 12 h, the migrating cells on the surface were fixed with 4% paraformaldehyde and then dyed with 0.5% crystal violet. Finally, counting the number of stained cells by using an inverted microscope. For the wound healing assay, HB cells were seeded into a 6-well plate until confluence reaches 100%. Scratching the cell layer was necessary by using A 200 μL pipette tip. After that, the cells were cultured for 48 h. Finally, photographs were taken using an inverted microscope**.**

### Immunohistochemical (IHC) analysis

The HB tissue samples were fixed by using 4% paraformaldehyde and then embedded in paraffin. After deparaffinization and rehydration of the section, the samples were soaked in 3% H2O2 and then sealed with 5%FBS at 37 ºC for 25 min. Rabbit NAT10 antibody (1:2000; Abcam) and Rabbit Ki67 antibody (1:2000; Abcam), and Rabbit G6PD antibody (1:1000; Abcam) were incubated at 4 ºC overnight, followed by Goat anti-Rabbit IgG (1:2000, Beyotime) as secondary antibody, incubated for 1h. The signals were analysed with a light microscope.

### RNA sequencing

RNA libraries were constructed from each experimental group, including three biological replicates per sample. The sequencing platform utilized for poly(A) and RNA isolation, library construction, and sequencing was the MGISEQ-2000RS. Gene expression profiles were analyzed based on read count data. Using SAMtools (v1.7) and HTSeq-count (v0.9.1), expression values and transcription levels for each gene were estimated. Differentially expressed genes were identified using DESeq2 (v1.30.1), where a significance threshold of P ≤ 0.05 and an absolute fold change of ≥ 1 were applied.

### ROS and GSH assay

According to the instructions of the manufacturer, ROS and GSH in HB cells was measured by using a kit. After the cells have fully adhered, incubate for 30 minutes following the addition of the corresponding reagents as specified in the manual. The next, using trypsin digested the cells and centrifuging them at 1000 rpm. Finally, the step was resuspending the cells using PBS and analyzing for fluorescence intensity using flow cytometry.

### Lipid drop detection

First, the HB cells on the cell slide were fixed with 4% paraformaldehyde for 15 min, then dyed with BODIPY 493/503 for 30 min. The next, the samples were dyed with DAPI for 6 min at 37 ºC, and then washed with PBS at least 3 times. Finally, the images were captured by confocal microscope and the lipid droplet content was quantitatively and statistically analyzed by ImageJ software.

### acRIP-seq and acRIP-qPCR

Anti-ac4C antibody or IgG were used to incubate RNA fragments, and ac4C modified RNA will be eluted using N-acetyl-cytidine sodium salt for ac4C enrichment analysis through qPCR.

### mRNA stability assay

After incubating HB cells with actinomycin D, RNA will be extracted from the HB cells, and RT-qPCR will be performed to calculate the half-life of G6PD mRNA to assess mRNA stability.

### Dual-luciferase assay

First, the cDNA fragment containing the NAT10 promoter region was amplified by PCR and then connected into the pGLO luciferase reporter plasmid. HB Cells with the silence of YAP1 were seeded in a 24-well plate and cultured overnight, followed by using Lipofectamine 3000 transfection of the recombinant construct into the cells. Finally, the kit was used to detect luciferase activity.

### Chromatin immunoprecipitation-qPCR assay

Starting with the EZ ChIP kit according to the instructions of the manufacturer. The HB cells are cross-linked and formed into balls, and the lytic supernatant is treated ultrasonically. Protein G beads, 10 μg anti-YAP1 antibody or 10 μg rabbit IgG antibody were used to incubate the supernatant to detect the pulled and purified DNA fragments.

### Animal studies

All animal experiments were conducted in accordance with the guidelines approved by the Animal Ethics Committee of Zhongshan Hospital, Fudan University (WYYY-AEC-2023-083). BALB/c nude mice (4-6 weeks old; 20.0 +/- 2.0g; Male) bought from Shanghai Jiesijie Laboratory animal Company, and kept under specific pathogen free conditions. Transfected with sh-NAT10 or sh-NC cells (2×10^5^ cells per mouse, 0.2mLPBS) were injected subcutaneously into the lower back of mice. The next important step was measuring tumor size every 7 days and tumor mass after 28 days. Using a vernier caliper measured the tumor volume size (mm^3^) and it was calculated as: volume=length×width^2^×0.5. When the tumor diameter reached 5mm, the mice were randomly divided into treatment group with 5 mice in every group. The mice were killed at 28 days, and the tumors were dissected. The paraffin-embedded sections were dewaxed and stained with H&E and IHC.

### Statistical analysis

GraphPad Prism 8 software was used for the statistical and analysis of the experiment results during the study. Data were expressed as mean ± standard, the experiments were statistically significant when *P* < 0.05. *P* values are indicated as follows: *(*p* < 0.05), **(*p* < 0.01) and *** (*p* < 0.001).

## Results

### NAT10 expression is upregulated in hepatoblastoma, promoting HB cell proliferation and metastasis *in vitro*

**Figure 1:**
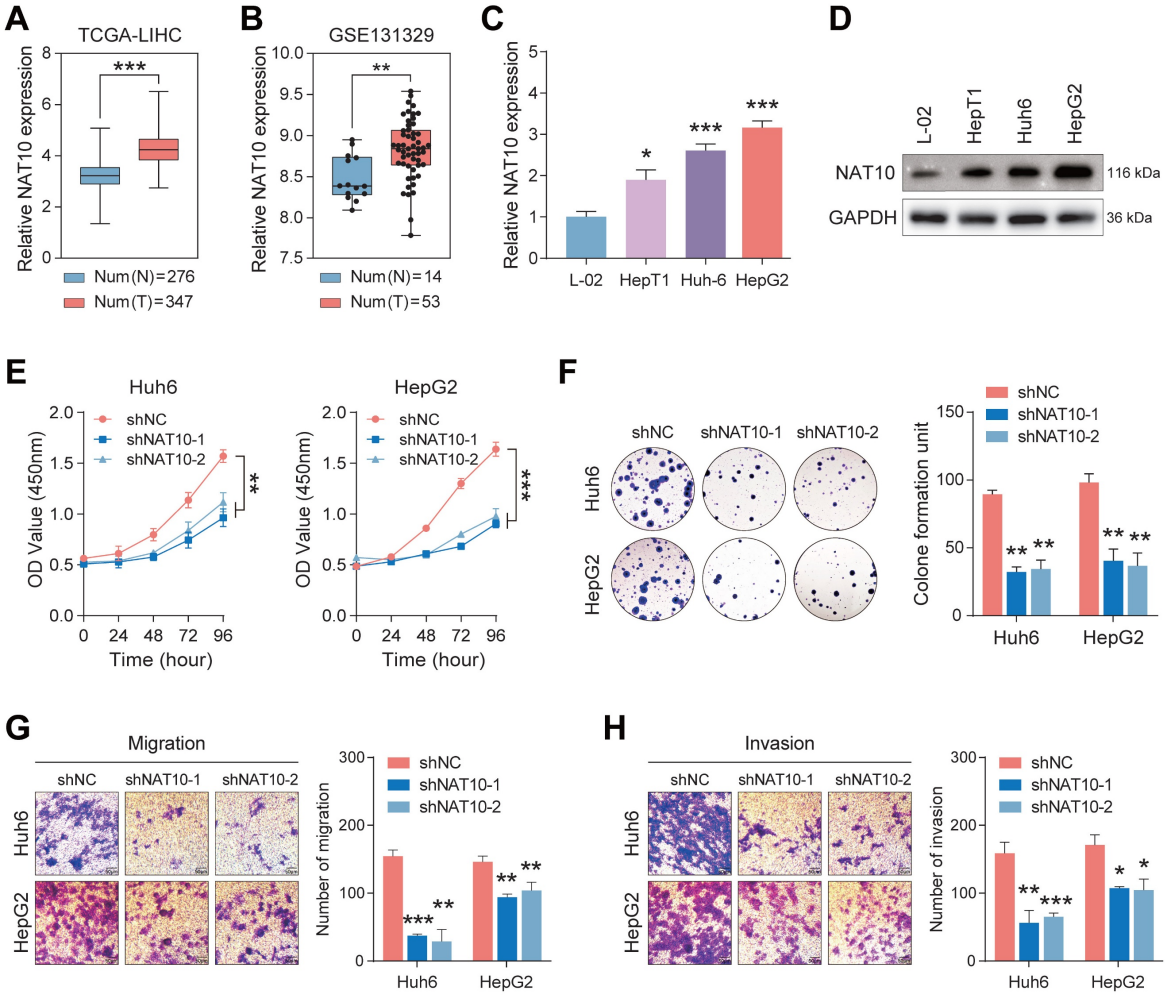
** NAT10 expression is upregulated in hepatoblastoma, promoting HB cell proliferation and metastasis.** (A) Validation of high NAT10 expression in HB using the TCGA database. (B) Prediction of high NAT10 expression in HB from the GSE131329 dataset. (C) qRT-PCR detected NAT10 expression in HB cell lines. (D) Western blot detected NAT10 protein expression levels in HB cell lines. (E-F) CCK-8 and the colony formation assays validated the decreased proliferation capability of NAT10-deficient HB cells. (G-H) The Transwell assays showed reduced migration and invasion capabilities in NAT10-deficient cells. Data information: In all relevant panels, ns, no significant; *P < 0.05; **P < 0.01; ***P < 0.001; ****P < 0.0001; two-tailed t-test. Data are presented as mean ±SD and are representative of three independent experiments.

To investigate NAT10 expression in hepatoblastoma (HB) tissues, we analyzed data from the TCGA and GSE131329 databases. The results demonstrated a significant increase in NAT10 expression in HB tissues (Fig. 1A, B). To validate these findings, we assessed NAT10 expression levels in HB cell lines using quantitative real-time polymerase chain reaction (qRT-PCR) and Western blot analysis. As anticipated, NAT10 expression was markedly elevated in HepT1, Huh6, and HepG2 cells (Fig. 1C, D). This indicated that NAT10 expression was significantly increased in HB tumor tissues and cell lines. We subsequently examined the role of NAT10 in HB cell proliferation and invasion by transfecting three independent shRNAs into Huh6 and HepG2 cells (Fig. S1A, B). CCK-8 and colony formation assays revealed that NAT10 knockdown significantly reduced the proliferative capacity of HB cells (Fig. 1E, F). Moreover, Transwell assays indicated that the invasion and migration capabilities of HB cells were markedly compromised following NAT10 knockdown (Fig. 1G, H). Collectively, these results suggest that NAT10 promotes HB cell proliferation and invasion *in vitro*.

### NAT10 promotes HB cell proliferation and metastasis *in vivo*

**Figure 2:**
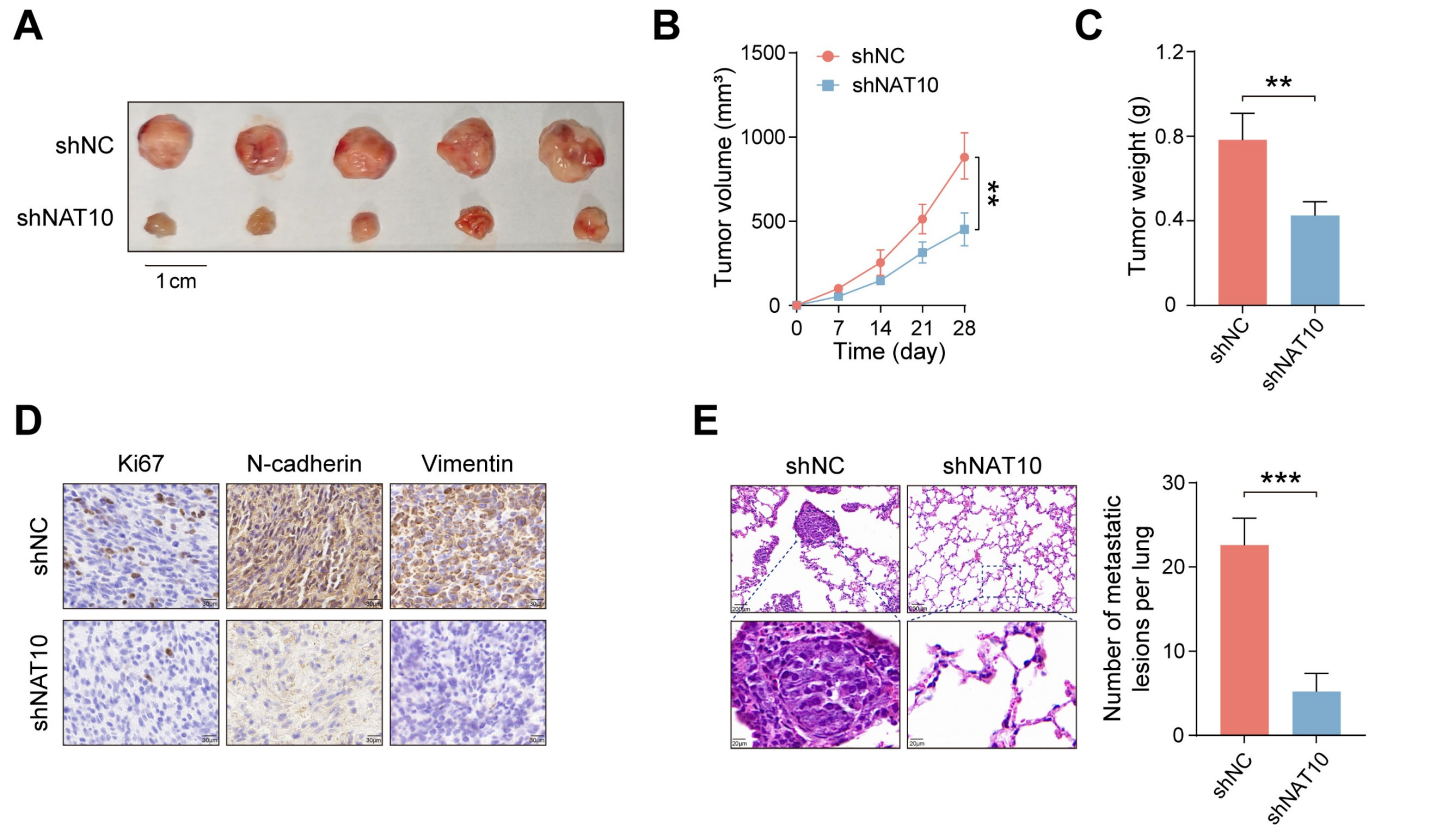
** NAT10 promotes HB cell proliferation and metastasis *in vivo*. (A)** Knockdown of NAT10 effectively inhibited the growth of subcutaneous HB tumors in nude mice. (B) Weekly monitoring of tumor volumes and plotting of tumor growth curves. (C) Tumors were excised and weighed after 28 days. (D) IHC staining of tumor sections showed significantly decreased expression of Ki-67, N-cadherin, and Vimentin in NAT10-deficient HB cells. (E) The number of lung metastases from HB tumors was significantly reduced following NAT10 knockdown. Data information: In all relevant panels, ns, no significant; *P < 0.05; **P < 0.01; ***P < 0.001; ****P < 0.0001; two-tailed t-test. Data are presented as mean ±SD and are representative of three independent experiments.

To further assess the tumorigenic potential of NAT10 *in vivo*, HB cell lines (shNC and shNAT10) were subcutaneously transplanted into nude mice. Our findings revealed that NAT10 knockdown significantly inhibited HB tumor growth (Fig. 2A). Tumor volumes were monitored weekly, with growth curves plotted accordingly. The results indicated that tumors from NAT10-knockdown groups were significantly smaller (Fig. 2B). On day 28, the mice were euthanized, and subcutaneous tumors were excised and weighed, confirming that NAT10 knockdown led to markedly reduced tumor weights (Fig. 2C). Notably, we performed immunohistochemical staining to further analyze the xenografted HB tumor tissues. The results indicated that Ki-67, N-cadherin, and Vimentin expression levels in HB tumor tissues were enormously lower when NAT10 was knocked down, indicating a marked reduction in the proliferation and invasion capabilities of HB cells (Fig. 2D). Interestingly, we also performed HE staining on the lung tissues of the nude mice, the number of lung metastases was notedly reduced following NAT10 knockdown (Fig. 2E). In summary, our findings indicated that NAT10 promoted HB cell proliferation and metastasis *in vivo*.

### NAT10 upregulates the pentose phosphate pathway (PPP) in HB

**Figure 3:**
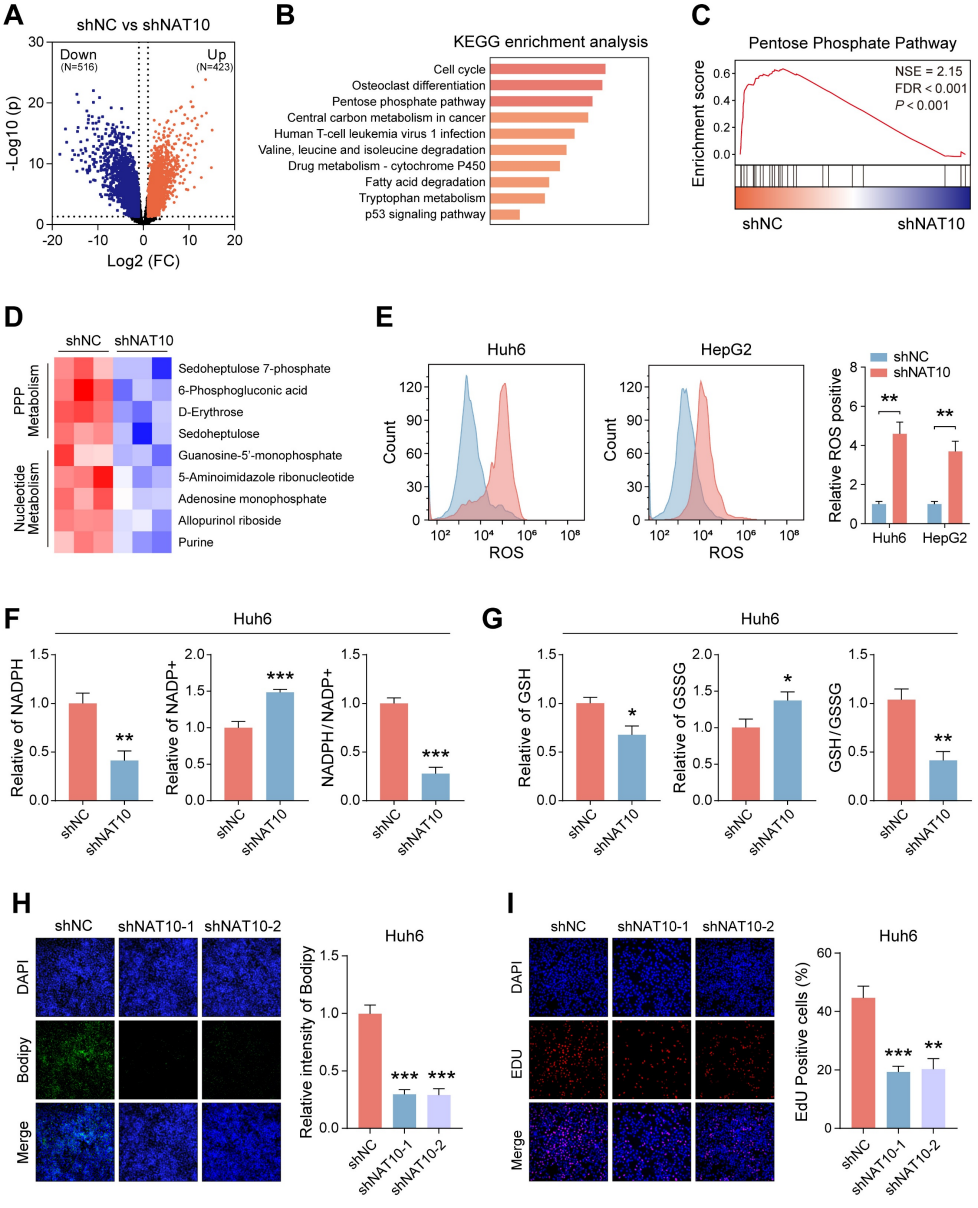
** NAT10 upregulates the pentose phosphate pathway in HB cells.** (A) Transcriptomic sequencing of NAT10-deficient HB cells and the corresponding volcano plot. (B-C) KEGG and GSEA pathway enrichment analysis. (D) Metabolomic sequencing of NAT10-deficient HB cells and the corresponding heatmap. (E) Flow cytometry analysis of ROS levels in NAT10-deficient HB cells. (F) Kit analysis of NADPH content and generation levels in NAT10-deficient HB cells. (G) Kit analysis of GSH generation levels in NAT10-deficient HB cells. (H) BODIPY analysis of lipid synthesis levels in NAT10-deficient HB cells. (I) EDU assay for measuring the proliferation capacity of NAT10-deficient HB cells. Data information: In all relevant panels, ns, no significant; *P < 0.05; **P < 0.01; ***P < 0.001; ****P < 0.0001; two-tailed t-test. Data are presented as mean ±SD and are representative of three independent experiments.

To further elucidate the role of NAT10 in HB cells, we conducted transcriptomic sequencing on Huh6 cells with NAT10 knockdown compared to control cells. The volcano plot illustrated that Huh6 cells with downregulated NAT10 exhibited downregulation of 516 genes and upregulation of 423 genes (Fig. 3A). KEGG and GSEA revealed that NAT10 knockdown primarily affected the pentose phosphate pathway (PPP) in HB cells (Fig. 3B, C). Given the significance of the PPP in cellular metabolism, we performed metabolomic sequencing on HB cells with and without NAT10. The heatmap indicated that NAT10 knockdown led to significant downregulation of specific metabolites in the PPP and nucleotide metabolism pathways (Fig. 3D).

Previous researches have indicated that PPP is a glucose oxidation pathway that serves as the main source of 5-phosphoribose and nicotinamide adenine dinucleotide phosphate (NADPH) 25. Furthermore, NADPH plays a crucial part in fatty acid synthesis and reactive oxygen species (ROS) clearance processes. Notably, NADPH is vital for the production of GSH, which is a major scavenger of ROS 26. To further verify whether NAT10 knockdown affected the PPP in HB cells, we measured the levels of ROS, NADPH, and GSH in HB cells. The results from the assay kits indicated that after NAT10 knockdown, the ROS levels significantly increased in both Huh6 and HepG2 cells, while the NADPH and GSH levels significantly decreased (Fig. 3E-G and Fig. S2A, B).

It is noteworthy that we also assessed the fatty acid level in HB cells using Bodipy staining. As expected, knocking down NAT10 significantly reduced fatty acid levels in Huh6 and HepG2 cells (Fig. 3H and Fig. S2C). Research indicates that excessive ROS accumulation can lead to cellular DNA damage. Subsequently, we assessed the proliferation capacity and DNA damage of HB cells using the EDU assay. The results indicated that knocking down NAT10 decreased the proliferation capacity and caused DNA damage in HB cells (Fig. 3I and Fig. S2D). We then treated the HB cells whose NAT10 was knocked down with the nucleophile Nuc and the reactive oxygen scavenger Nac. The results indicated that combined treatment with Nuc and Nac resulted in significantly higher viability in HB cells compared to either single treatment or untreated groups (Fig. S2E, F). Overall, these findings further confirmed that knocking down NAT10 affected the PPP in HB cells, thereby weakening the cell's proliferation ability.

### NAT10 mediates ac4C modification to upregulate G6PD expression

**Figure 4:**
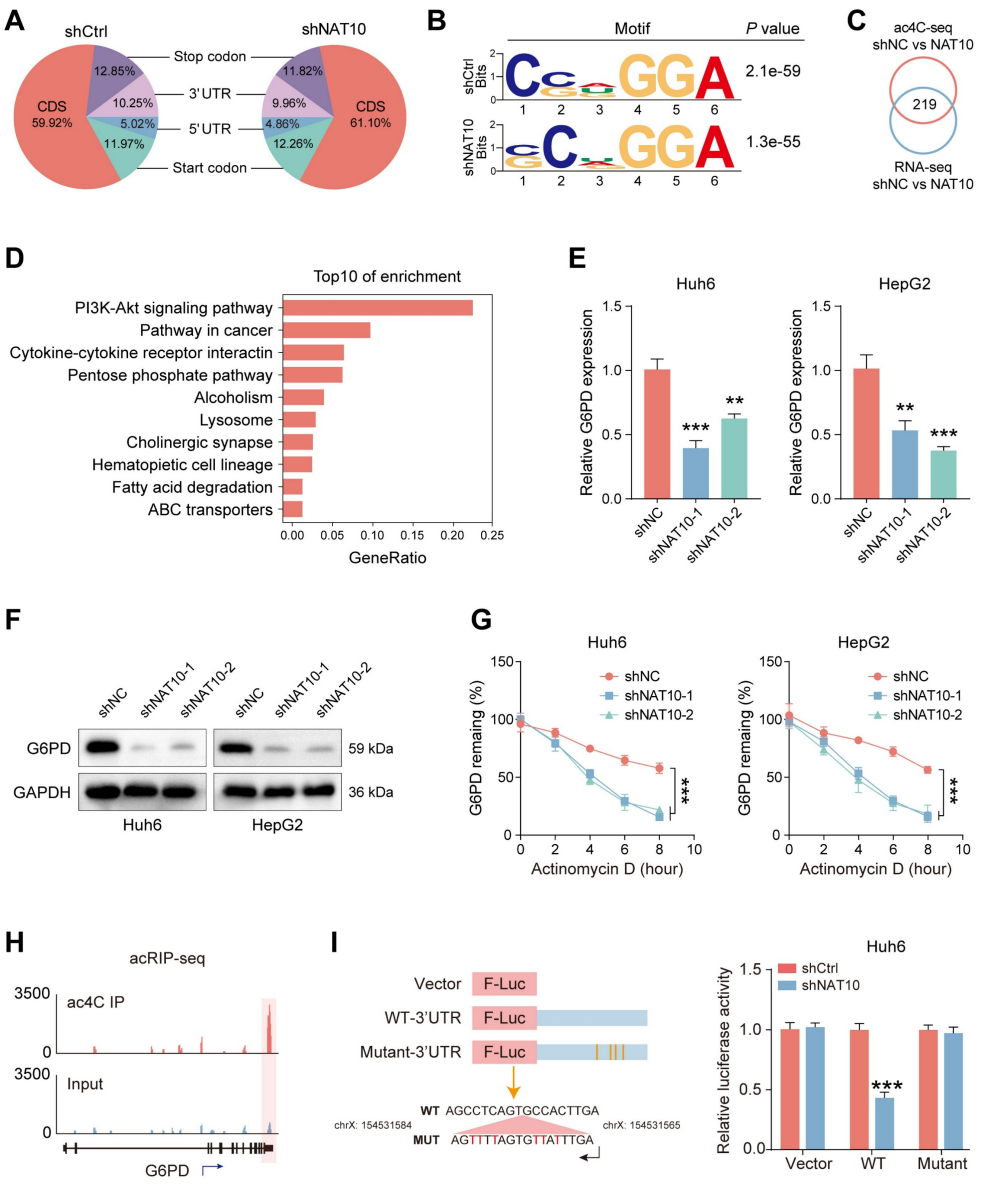
** NAT10 mediates ac4C modification to upregulate G6PD expression.** (A) Pie chart of ac4C distribution. (B) Motif description. (C) Venn diagram of intersecting ac4C sequencing and transcriptome sequencing. (D) KEGG pathway enrichment analysis of the intersecting genes. (E-F) qRT-PCR and Western blot detected G6PD expression after NAT10 knockdown. (G) Decreased stability of G6PD mRNA after NAT10 knockdown. (H) Peak graph of ac4C modification. (I) Dual-luciferase assay detected fluorescence intensity after NAT10 knockdown. Data information: In all relevant panels, ns, no significant; *P < 0.05; **P < 0.01; ***P < 0.001; ****P < 0.0001; two-tailed t-test. Data are presented as mean ±SD and are representative of three independent experiments.

The recent study has shown that NAT10 mediated ac4C mRNA acetylation in hepatocellular carcinoma (HCC) 27. To further investigate the genes which took part in ac4C modification in HB, we conducted acRIP-seq on HB cells whose NAT10 was knocked down and the control cells. The results indicated that when RNA was categorized into mRNA coding sequences (CDS), 3' UTR, 5' UTR regions, and non-coding RNAs, the distribution patterns of ac4C in control HB cells and NAT10 knockdown HB cells exhibited similarity (Fig. 4A). Additionally, the ac4C motif "CXX" was enriched in the identified peaks (Fig. 4B). The next, we conducted a combined analysis of acRIP-seq and RNA-seq results, identifying a total of 219 differentially expressed genes (Fig. 4C). We performed KEGG pathway enrichment analysis on the intersecting genes, which revealed significant changes in the PPP pathway in HB cells after knocking down NAT10 (Fig. 4D). It is well-known that G6PD is an important enzyme in the PPP pathway that is crucial for NADPH generation 28. We then assessed G6PD expression in HB cells post-NAT10 knockdown using qRT-PCR and Western blot analysis. The results showed that G6PD expression was reduced in HB cells after NAT10 knockdown (Fig. 4E, F). Furthermore, we treated HB cells with radiolabeled actinomycin D at various time points to analyze the mRNA stability of G6PD through qRT-PCR. As expected, G6PD mRNA stability was significantly decreased following NAT10 knockdown in HB cells (Fig. 4G).

To further examine how NAT10 affects G6PD expression in HB cells, we conducted a detailed analysis of the acRIP-seq results. The peak graph indicated that the ac4C enrichment of G6PD was significantly reduced in HB cells whose NAT10 was knocked down (Fig. 4H). In addition, the luciferase assay indicated that knocking down NAT10 led to a reduction in the luciferase activity within the ac4C peak region of G6PD mRNA, while no such attenuation was observed in the cytidine-mutated region (Fig. 4I and Fig.S3C). To validate whether G6PD mRNA is a direct target of ac4C modification in HB cells, we also performed acRIP-qPCR and the results were consistent with previous expectations, G6PD ac4C modification levels were significantly lower in HB cells after NAT10 knockdown (Fig. S3A). Notably, analysis of the TCGA database and HB tumor tissue samples revealed a positive relevance between NAT10 and G6PD expression (Fig. S3B). In summary, these findings indicate that in HB, NAT10 mainly enhances G6PD expression through ac4C modification.

### NAT10 promotes the malignant progression of HB by upregulating the G6PD-dependent PPP

**Figure 5:**
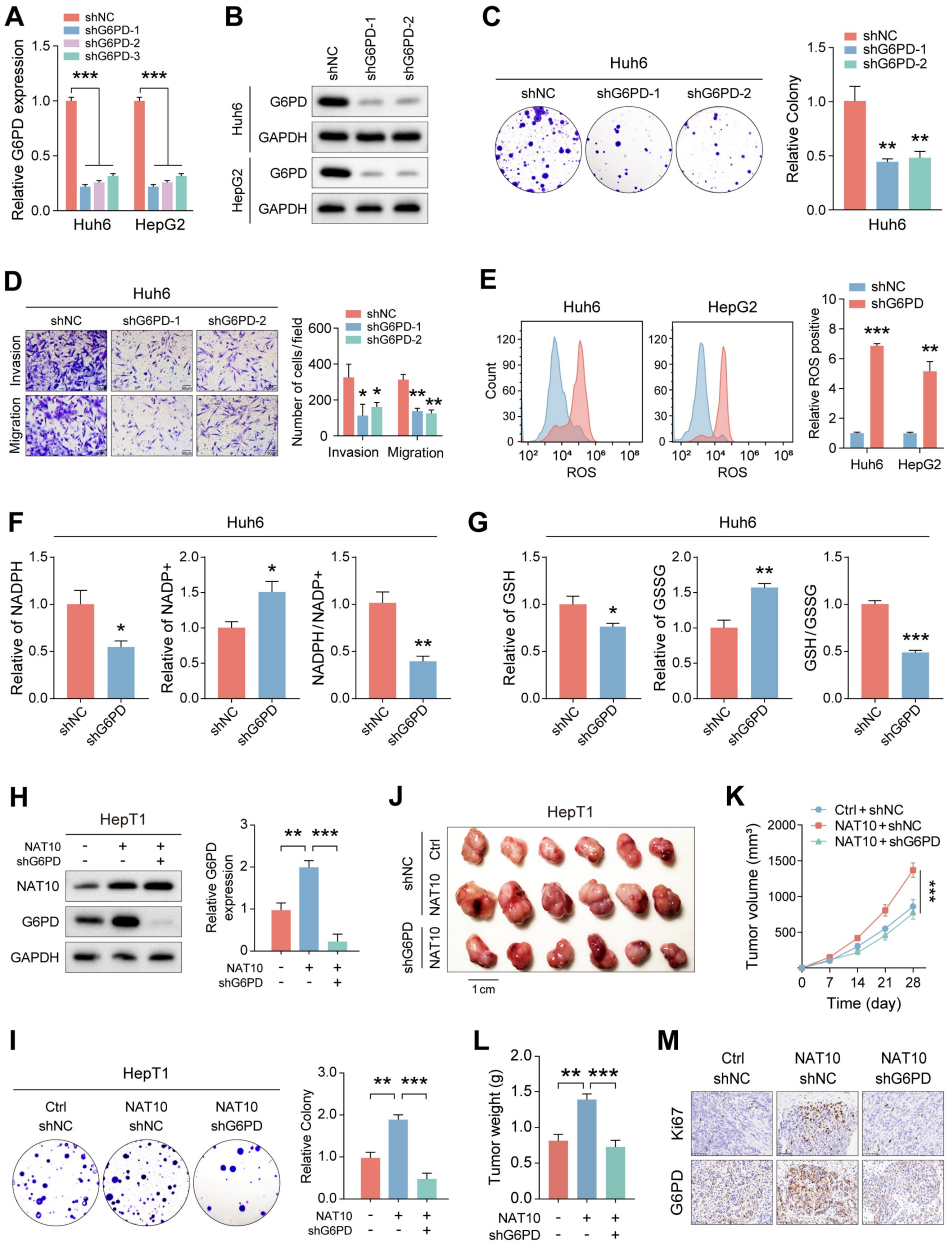
** NAT10 promotes the malignant progression of HB by upregulating the G6PD-dependent PPP pathway. (A-B)** qRT-PCR and Western blot detected analyses of G6PD knockdown effects. (C-D) The colony formation and Transwell assays assessed the proliferation and migration capabilities of G6PD-deficient HB cells. (E-G) Detection of ROS levels, NADPH content and generation levels, and GSH generation levels in G6PD-deficient HB cells using kits. (H) Western blot and qRT-PCR detected G6PD expression levels after NAT10 overexpression and G6PD knockdown. (I) The colony formation assay showed that G6PD knockdown suppresses the increase in proliferation ability induced by NAT10 overexpression. (J-L) G6PD knockdown inhibit ed the enhanced tumor growth resulting from NAT10 overexpression. (M) Immunohistochemistry examining the expressions of G6PD and Ki-67. Data information: In all relevant panels, ns, no significant; *P < 0.05; **P < 0.01; ***P < 0.001; ****P < 0.0001; two-tailed t-test. Data are presented as mean ±SD and are representative of three independent experiments.

To ensure the successful execution of subsequent functional experiments, we established stable G6PD knockdown cell lines in Huh6 and HepG2 cells (Fig. 5A, B). We then assessed the impact of G6PD on the functionality of HB cells by evaluating the proliferation and invasion levels of the G6PD-deficient HB cells. The results indicated that G6PD knockdown significantly reduced the proliferation and invasion capabilities of HB cells, indicating the critical role of G6PD in HB progression (Fig. 5C, D, and Fig. S4A-D). Next, to explore changes in the PPP in G6PD-deficient HB cells, we utilized kits to measure ROS levels, NADPH content, and GSH generation in the HB cells.

Notably, following G6PD knockdown, ROS levels significantly increased, while NADPH content and generation, as well as GSH production, were markedly decreased (Fig. 5E-G and Fig. S4E, F). These findings suggest significant inhibition of the PPP pathway in G6PD-deficient cells.

To further investigate the association between NAT10 and G6PD in HB cells, we attempted to knock down G6PD in HepT1 cells overexpressing NAT10. The result indicated that overexpression of NAT10 significantly enhanced the G6PD expression in HepT1 cells using Western blot (Fig. 5H). Furthermore, after G6PD knockdown, G6PD expression of in NAT10-overexpressing HepT1 cells was significantly downregulated (Fig. 5H). Subsequently, we assessed the proliferation and invasion levels of G6PD-deficient HepT1 cells with NAT10 overexpression. The results indicated that the number of colonies formed by HepT1 cells was significantly lower in the G6PD-deficient with NAT10 overexpression group, indicating a marked reduction in cell proliferation ability. Furthermore, when compared to the group with NAT10 overexpressed, the number of invasive and migratory cells of of G6PD-deficient with NAT10 overexpression was markedly decreased (Fig. 5I and Fig. S4G). Furthermore, when NAT10 was simultaneously overexpressed and G6PD was downregulated, the levels of ROS significantly decreased, while the content of NADPH and GSH increased markedly (Fig. S4H-I).

Next, to study the tumorigenic capacity of G6PD *in vivo*, we subcutaneously transplanted HepT1 cell-shNC, HepT1 cell-NAT10, and HepT1 cell-NAT10-shG6PD into nude mice and monitored the tumor sizes timely (Fig. 5J). We also measured tumor volumes every seven days and plotted tumor growth curves. Ultimately, on day 28, we sacrificed the nude mice and excised the tumor tissues for weighing. The results revealed that knocking down G6PD significantly inhibited the promoting effect of NAT10 overexpression on tumor growth in the nude mice (Fig. 5K, L). Notably, these results indicated that G6PD could promote the proliferation, invasion, and migration abilities of HB cells both *in vitro* and *in vivo*. Finally, we conducted immunohistochemical staining to further analyze the HB tumor tissues. The results indicated that in tumor tissues with NAT10 overexpression and G6PD knockdown, the G6PD and Ki-67 expression were significantly lower (Fig. 5M). In summary, we found that NAT10 primarily promotes the occurrence and progression of HB by upregulating the G6PD-dependent PPP.

### YAP1 regulates NAT10 expression and activates PPP, thereby promoting malignant progression of HB

**Figure 6:**
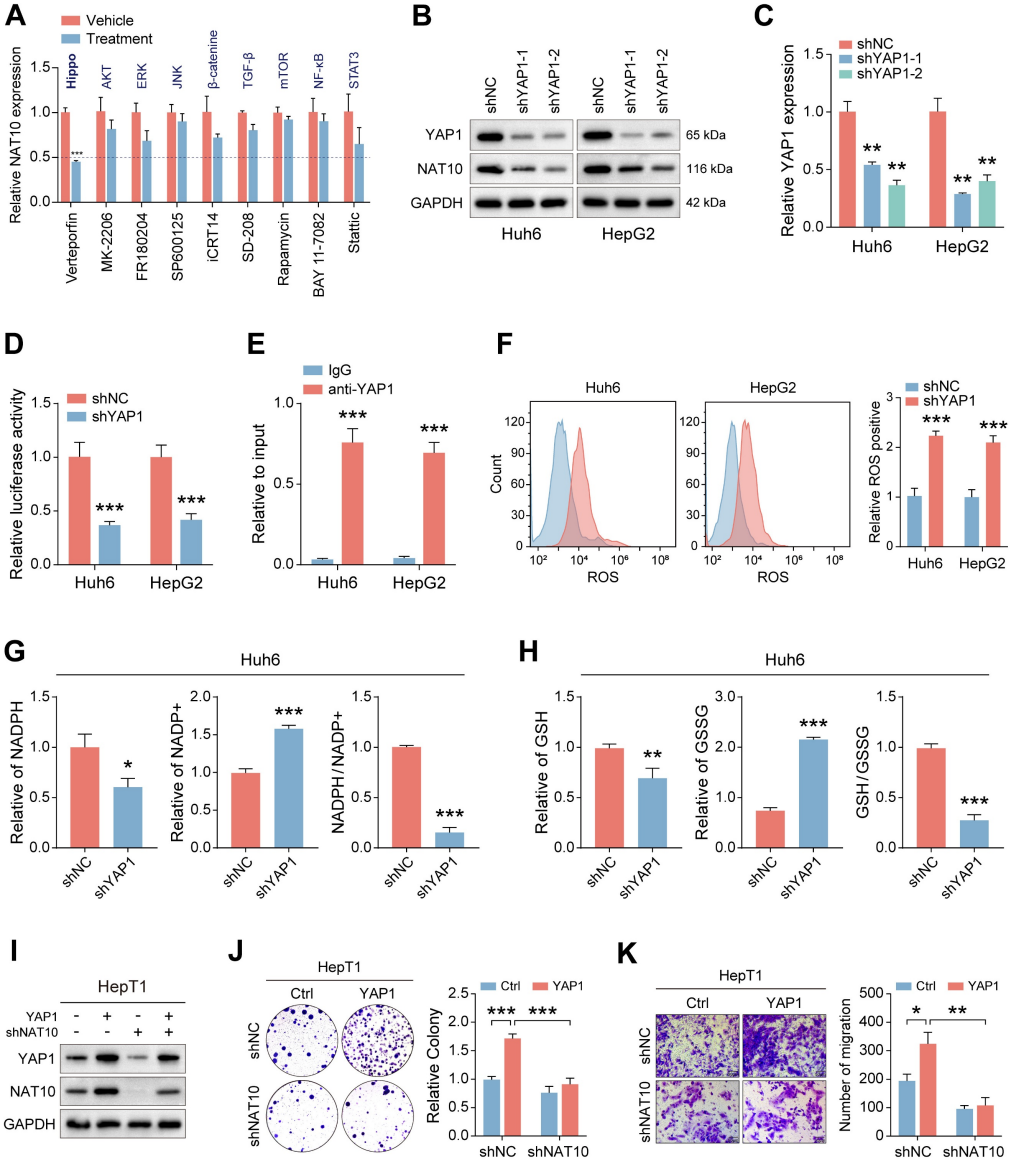
** YAP1 regulates NAT10 expression and activates PPP, thereby promoting malignant progression of HB.** (A) Measurement of NAT10 expression levels in HB cells treated with different small molecule inhibitors of signaling pathways using qRT-PCR. (B-C) Western blot and qRT-PCR detected the effectiveness of YAP1 knockdown. (D-E) Dual-luciferase reporter assay and ChIP-PCR demonstrated that YAP1 binded to the NAT10 promoter. (F-H) Kit assays detected ROS levels, NADPH content and generation levels, and GSH generation levels in YAP1-deficient HB cells. (I) Western blot detected NAT10 and YAP1 expression levels after NAT10 knockdown and YAP1 overexpression. (J-K) The colony formation and Transwell assays showed that NAT10 knockdown inhibited the enhancement of proliferation and migration induced by YAP1 overexpression. Data information: In all relevant panels, ns, no significant; *P < 0.05; **P < 0.01; ***P < 0.001; ****P < 0.0001; two-tailed t-test. Data are presented as mean ±SD and are representative of three independent experiments.

To elucidate potential upstream signaling pathways regulating NAT10 expression, we treated hepatoblastoma (HB) cells with various small-molecule inhibitors and assessed NAT10 expression via qRT-PCR. The data indicated that inhibiting YAP1 significantly reduced NAT10 expression, suggesting that the YAP1 signaling pathway may regulate NAT10 (Fig. 6A). To further validate this hypothesis, we utilized two independent shRNAs to knock down YAP1 in Huh6 and HepG2 cells. Successful knockdown was confirmed by significantly decreased YAP1 expression relative to control groups (Fig. 6B, C). Cell proliferation assays demonstrated that YAP1-deficient HB cells exhibited markedly reduced proliferative capability, underscoring the importance of YAP1 in HB growth (Fig. S5A, B). Additionally, wound healing assays revealed decreased migration rates and distances for YAP1 knockdown cells compared to controls (Fig. S5C).

To further investigate the association between YAP1 and NAT10, we successfully constructed a dual-luciferase reporter vector containing the NAT10 promoter. The results indicated that the fluorescence intensity of HB cells whose YAP1 was knocked down decreased significantly, indicating a dramatic reduction in NAT10 expression (Fig. 6D). Additionally, we performed ChIP-qPCR and found that YAP1 could bind to the NAT10 promoter, further supporting the idea that YAP1 regulates NAT10 expression (Fig. 6E). Next, we aimed to explore whether YAP1 knockdown would affect the PPP in HB cells. We used kits to measure the levels of ROS, NADPH content and generation and GSH generation to observe changes in the PPP. The results indicated that ROS levels were significantly elevated in YAP1-deficient HB cells, while NADPH content and generation level and GSH generation level were all significantly decreased, suggesting that the YAP1 signaling pathway is critical for the PPP in HB cells (Fig. 6F-H and Fig. S5D, E). Notably, we conducted experiments on NAT10-deficient HepT1 cells with YAP1 overexpression, followed by Western blot to assess changes in the expression of NAT10 and YAP1. The results indicated that YAP1 overexpression could restore YAP1 and NAT10 expression in NAT10-deficient HepT1 cells, indicating that YAP1 is very important for NAT10 (Fig. 6I). Additionally, we performed cell proliferation and invasion experiments using NAT10-deficient HepT1 cells with YAP1 overexpression. The results indicated that overexpressing YAP1 enhanced the proliferative and invasive capacity of HB cells, but knocking down NAT10 inhibited the enhancement of cell proliferation and invasion induced by YAP1 overexpression (Fig. 6J, K). Besides, when NAT10 was simultaneously overexpressed and G6PD was downregulated, the levels of ROS significantly decreased, while the content of NADPH and GSH increased markedly (Fig. S5G-I). Overall, YAP1 is crucial for the expression of NAT10 in HB cells and acts as a potential upstream signal regulating its expression.

### The NAT10 inhibitor Remodelin effectively inhibits the malignant progression of HB

**Figure 7:**
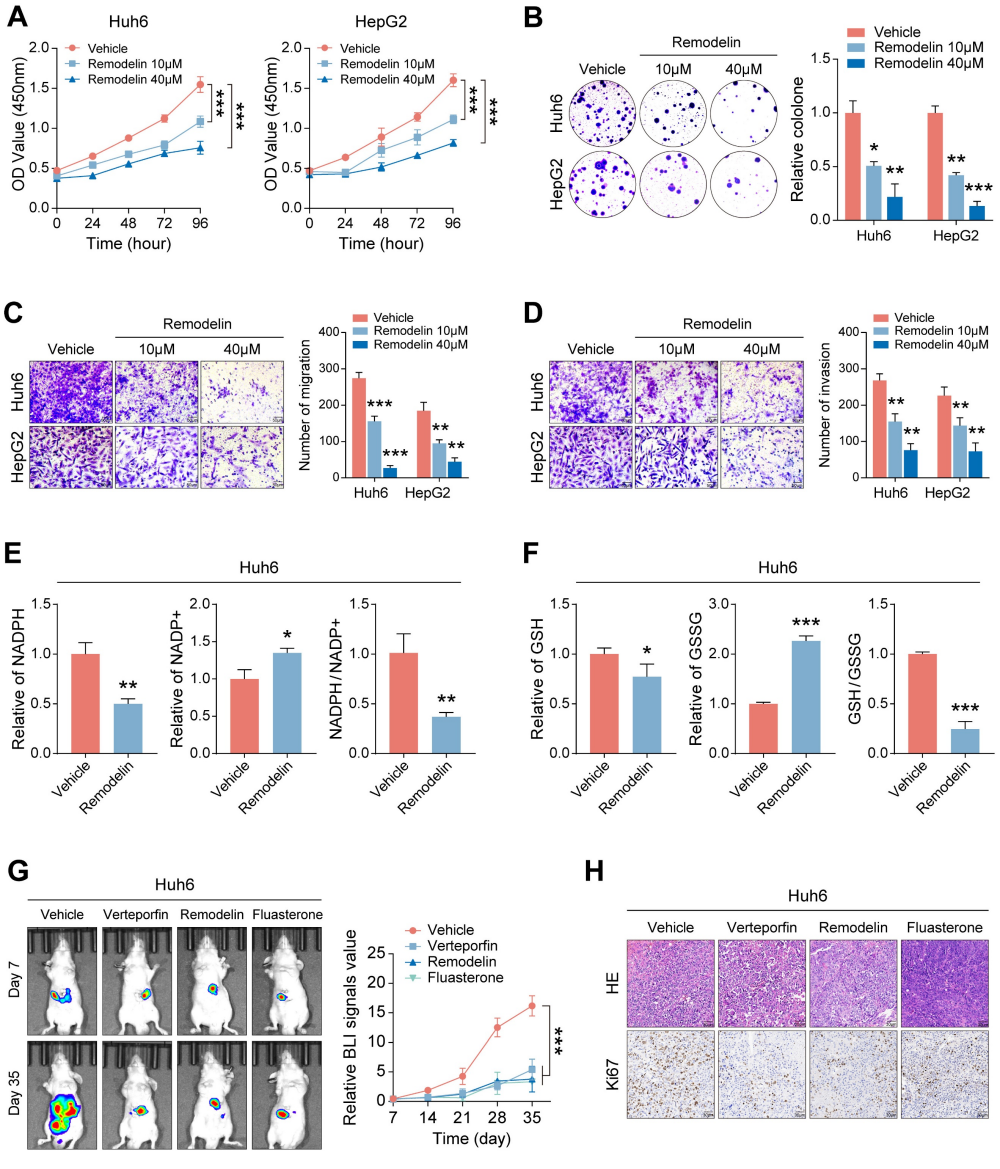
** The NAT10 inhibitor Remodelin effectively inhibits the malignant progression of HB. (A-B)** The cell proliferation assays showed that Remodelin inhibited cell proliferation capacity. (C-D) The Transwell assay showed that Remodelin inhibited cell invasion and migration capacity. (E-F) Kits detected NADPH content and generation levels, and GSH generation levels in HB cells treated with Remodelin. (G-H) In situ tumor experiments and immunohistochemistry showed that Remodelin effectively inhibited tumor growth in nude mice. Data information: In all relevant panels, ns, no significant; *P < 0.05; **P < 0.01; ***P < 0.001; ****P < 0.0001; two-tailed t-test. Data are presented as mean ±SD and are representative of three independent experiments.

The observed proliferation-promoting role of NAT10 in HB cells suggests its potential as a therapeutic target. To evaluate this, we treated HB cells with varying concentrations of the NAT10 inhibitor Remodelin and assessed cell proliferation. The results demonstrated that Remodelin significantly inhibited HB cell proliferation (Fig. 7A, B and Fig. S6A, B). Additionally, Transwell assays indicated that Remodelin treatment markedly impaired HB cell invasion and migration (Fig. 7C, D). Overall, the NAT10 inhibitor Remodelin significantly suppressed the proliferation, invasion, and metastatic abilities of HB cells *in vitro*. In addition, our research has shown that NAT10 upregulated the PPP in HB cells. To further validate the impact of Remodelin on the PPP of HB cells, we used kits to measure NADPH content and generation level and GSH generation level in HB cells. Notably, Remodelin significantly reduced the NADPH content and GSH generation level in HB cells, indicating that Remodelin could suppress the PPP in HB cells to some extent (Fig. 7E, F and Fig. S6C, D).

Lastly, we validated the therapeutic efficacy of Remodelin *in vivo*. Huh6 cells were subcutaneously transplanted into nude mice, which were monitored weekly for tumor size while receiving treatments with the YAP1 inhibitor Verteporfin, the NAT10 inhibitor Remodelin, and the G6PD inhibitor Fluasterone. All three inhibitors exhibited significant therapeutic effects, markedly inhibiting tumor growth (Fig. 7G). Notably, we performed HE staining and immunohistochemistry for further analysis of the HB tumor tissues. As expected, the Ki-67 level in the HB tumor tissues of nude mice treated with Remodelin was significantly reduced, suggesting that the NAT10 inhibitor has good therapeutic potential *in vivo* (Fig. 7H). Moreover, we assessed the toxicity of remodeline in various human normal cell types, and the results indicated that, within a certain concentration range, remodeline does not exert significant toxic side effects on humans (Fig. 6E).

In conclusion, our findings establish NAT10 as an effective therapeutic target for HB, with Remodelin demonstrating promising potential to inhibit malignant progression, thereby providing a novel strategy for HB treatment.

## Discussion

HB is a common malignant liver tumor in children, often occurring in infants under 4 years of age 29. Currently, treatment strategies primarily include surgery and chemotherapy, sometimes combined with liver transplantation 3. However, the side effects of chemotherapy are significant, and prognosis for patients is poor, highlighting the urgent need for effective and less harmful treatment options. Metabolic reprogramming is an important marker of cancer progression, facilitating cancer cell growth, energy acquisition, and utilization 3. Therefore, studying the metabolic characteristics of HB cells may lead to new drug targets and treatment strategies, representing a new trend in research.

Previous researches have indicated that NAT10 plays a significant part in various malignant tumors, including cervical cancer 14, gastric cancer 13, and bladder cancer 12. NAT10 is highly expressed in multiple cancers and primarily takes part in several cellular biological processes through its acetyltransferase activity 13, 30. In this study, we found that NAT10 plays a critical part in the metabolic reprogramming of cancer cells. Through clinical data analysis and both *in vitro* and *in vivo* experiments, we observed that NAT10 significantly promotes the occurrence and progression of HB. Notably, analyses of transcriptomic and metabolomic sequencing results from HB cells with NAT10 knockdown indicated that NAT10 was crucial for the PPP in cancer cells. PPP is an alternative metabolic pathway to glycolysis that not only provides nucleotide precursors necessary for cell proliferation but also generates substantial NADPH for cellular lipid synthesis and antioxidant defense 31, 32. In addition, using a detection kit, we observed that the ROS levels in NAT10-deficient HB cells increased, while the generation of NADPH and GSH was significantly reduced, further validating our sequencing results. In summary, our research demonstrates that NAT10 promotes the proliferation of HB cells by activating PPP.

The ac4C modification is a highly conserved RNA modification, and NAT10 is currently known as the only enzyme capable of catalyzing the ac4C modification 10, 33. By jointly analyzing ac4C sequencing and transcriptomic sequencing results, we found that genes overlapping in these analyses were primarily enriched in the PPP through KEGG pathway enrichment analysis. G6PD is an important rate-limiting enzyme in the PPP, essential for the production of ribose and NADPH 34. G6PD is overexpressed in different cancers, including gastric cancer 35, bladder cancer 36, and breast cancer 37. However, studies on G6PD in HB are limited. Notably, we discovered that knocking down NAT10 in HB cells significantly reduced G6PD expression. Furthermore, the ac4C modification level and stability of G6PD mRNA were reduced in NAT10-deficient HB cells, suggesting that G6PD may be a direct target gene of NAT10.

Previous researches have shown that NAT10 inhibits ferroptosis by stabilizing the mRNA of ferroptosis suppressor protein 1 (FSP1) through ac4C modification, promoting the progression of colorectal cancer 38. This indicates that NAT10-mediated ac4C modification is conducive to cancer occurrence and progression. Additionally, we validated that NAT10 expression is positively correlated with G6PD expression by using the TCGA database and clinical tissue samples. Consistent with the results from NAT10 knockdown in HB cells, we found that ROS levels in G6PD-deficient HB cells increased, and the generation of NADPH and GSH was significantly reduced, indicating that the PPP was inhibited. Interestingly, knocking down G6PD inhibited HB tumor growth both *in vitro* and *in vivo*. Thus, our results suggest that NAT10 activates the PPP and promotes malignant progression of HB by upregulating the expression level of G6PD through mediating ac4C modification.

YAP is a major effector of the Hippo tumor suppressor pathway and is crucial for cell growth, maintenance of tissue homeostasis, and organ development 39. YAP1 is overexpressed in multiple cancers and plays an important part in promoting epithelial-mesenchymal transition, invasion, and metastasis of cancer cells 40, 41. Our study found that treatment of HB cells with the YAP1 inhibitor Verteporfin significantly reduced the expression of NAT10. We further validated the interaction between YAP1 and the NAT10 promoter using dual-luciferase assays and ChIP-qPCR. Interestingly, the overexpression of YAP1 effectively restored NAT10 expression in NAT10-deficient HB cells. Additionally, we discovered that the PPP was suppressed in YAP1-deficient HB cells. Previous studies have indicated that YAP and TAZ are involved in metabolic regulatory processes, such as promoting glycolysis and glutaminolysis, and their expression is also regulated by substances like glucose and fatty acids 24, 42, 43. Finally, we confirmed that the Verteporfin could inhibit the proliferation of HB cells and tumor growth to some extent, both *in vitro* and *in vivo*. Overall, our research indicates that NAT10 expression is regulated by the YAP1, and that the YAP1 inhibitor Verteporfin has a significant effect on HB treatment.

## Conclusions

**Figure 8:**
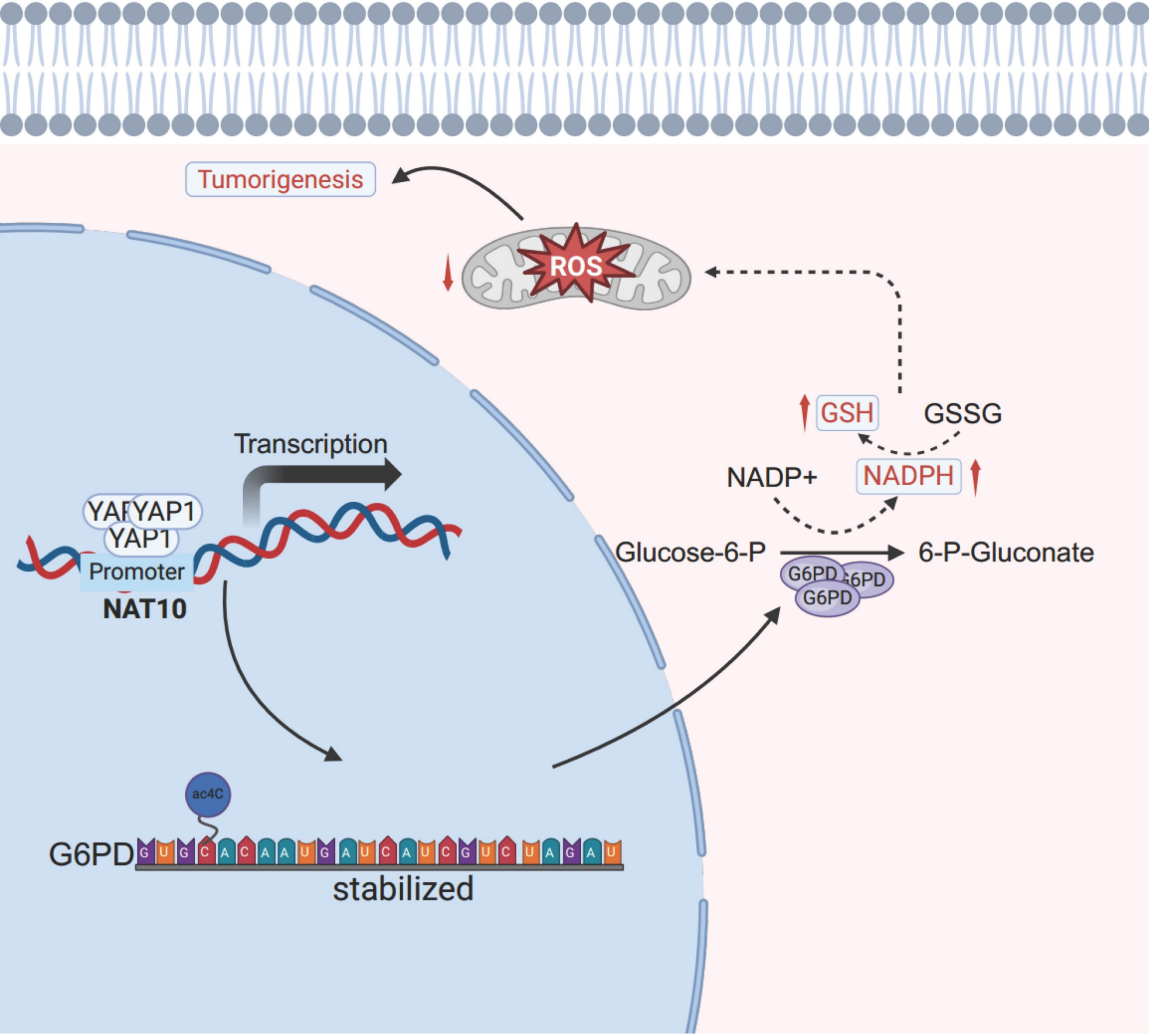
The schematic diagram depicts the regulation of the YAP1/NAT10/G6PD axis on tumor cells.

In summary, our study reveals that NAT10 is regulated by the upstream signal YAP1 and promotes the malignant progression of HB by mediating ac4C modification to upregulate the expression of G6PD and activate the PPP (Fig 8). Additionally, the NAT10 inhibitor Remodelin significantly inhibits the proliferation capability of HB cells both *in vitro* and *in vivo*. Therefore, our findings underscore the potential of targeting NAT10 as an important treatment strategy for HB, providing new insights for overcoming resistance and developing novel therapeutic approaches.

## Supplementary Material

Supplementary figures.

## Abbreviations

HB: Hepatoblastoma
PPP: Pentose phosphate pathway
NAT10: N-acetyltransferase 10
ac4C: N4-acetylcytidine
NADPH: Nicotinamide adenine dinucleotide phosphate
ROS: Reactive oxygen species
G6PD: Glucose-6-phosphate dehydrogenase
YAP1: Yes-associated protein 1)
FSP1: Ferroptosis suppressor protein 1

## References

[1] Clavería-Cabello A, Herranz JM, Latasa MU, Arechederra M, Uriarte I, Pineda-Lucena A (2023). Identification and experimental validation of druggable epigenetic targets in hepatoblastoma. J Hepatol.

[2] Wu PV, Rangaswami A (2022). Current Approaches in Hepatoblastoma-New Biological Insights to Inform Therapy. Curr Oncol Rep.

[3] Fu Y, Francés R, Monge C, Desterke C, Marchio A, Pineau P (2024). Metabolic and Epigenetic Mechanisms in Hepatoblastoma: Insights into Tumor Biology and Therapeutic Targets. Genes (Basel).

[4] Cao Y, Wu S, Tang H (2024). An update on diagnosis and treatment of hepatoblastoma. Biosci Trends.

[5] Zsiros J, Brugieres L, Brock P, Roebuck D, Maibach R, Zimmermann A (2013). Dose-dense cisplatin-based chemotherapy and surgery for children with high-risk hepatoblastoma (SIOPEL-4): a prospective, single-arm, feasibility study. Lancet Oncol.

[6] Arango D, Sturgill D, Alhusaini N, Dillman AA, Sweet TJ, Hanson G (2018). Acetylation of Cytidine in mRNA Promotes Translation Efficiency. Cell.

[7] Bartee D, Nance KD, Meier JL (2022). Site-Specific Synthesis of N(4)-Acetylcytidine in RNA Reveals Physiological Duplex Stabilization. J Am Chem Soc.

[8] Luo J, Cao J, Chen C, Xie H (2023). Emerging role of RNA acetylation modification ac4C in diseases: Current advances and future challenges. Biochem Pharmacol.

[9] Wei W, Zhang S, Han H, Wang X, Zheng S, Wang Z (2023). NAT10-mediated ac4C tRNA modification promotes EGFR mRNA translation and gefitinib resistance in cancer. Cell Rep.

[10] Rodrigues P, Bangali H, Ali E, Nauryzbaevish AS, Hjazi A, Fenjan MN (2024). The mechanistic role of NAT10 in cancer: Unraveling the enigmatic web of oncogenic signaling. Pathol Res Pract.

[11] Xie L, Zhong X, Cao W, Liu J, Zu X, Chen L (2023). Mechanisms of NAT10 as ac4C writer in diseases. Mol Ther Nucleic Acids.

[12] Xie R, Cheng L, Huang M, Huang L, Chen Z, Zhang Q (2023). NAT10 Drives Cisplatin Chemoresistance by Enhancing ac4C-Associated DNA Repair in Bladder Cancer. Cancer Res.

[13] Zhang Y, Jing Y, Wang Y, Tang J, Zhu X, Jin WL (2021). NAT10 promotes gastric cancer metastasis via N4-acetylated COL5A1. Signal Transduct Target Ther.

[14] Chen X, Hao Y, Liu Y, Zhong S, You Y, Ao K (2023). NAT10/ac4C/FOXP1 Promotes Malignant Progression and Facilitates Immunosuppression by Reprogramming Glycolytic Metabolism in Cervical Cancer. Adv Sci (Weinh).

[15] Ahamed A, Hosea R, Wu S, Kasim V (2023). The Emerging Roles of the Metabolic Regulator G6PD in Human Cancers. Int J Mol Sci.

[16] Yang HC, Wu YH, Yen WC, Liu HY, Hwang TL, Stern A (2019). The Redox Role of G6PD in Cell Growth, Cell Death, and Cancer. Cells.

[17] Jiang P, Du W, Wu M (2014). Regulation of the pentose phosphate pathway in cancer. Protein Cell.

[18] Luo X, Wei M, Li W, Zhao H, Kasim V, Wu S (2023). PBX3 promotes pentose phosphate pathway and colorectal cancer progression by enhancing G6PD expression. Int J Biol Sci.

[19] Whitburn J, Rao SR, Morris EV, Tabata S, Hirayama A, Soga T (2022). Metabolic profiling of prostate cancer in skeletal microenvironments identifies G6PD as a key mediator of growth and survival. Sci Adv.

[20] Kodaka M, Hata Y (2015). The mammalian Hippo pathway: regulation and function of YAP1 and TAZ. Cell Mol Life Sci.

[21] Qadir J, Riaz SK, Taj K, Sattar N, Sahar NE, Khan JS (2021). Increased YAP1 expression is significantly associated with breast cancer progression, metastasis and poor survival. Future Oncol.

[22] Liu X, Chen B, Xie F, Wong KY, Cheung AHK, Zhang J (2024). FOXP4 Is a Direct YAP1 Target That Promotes Gastric Cancer Stemness and Drives Metastasis. Cancer Res.

[23] Ajani JA, Xu Y, Huo L, Wang R, Li Y, Wang Y (2021). YAP1 mediates gastric adenocarcinoma peritoneal metastases that are attenuated by YAP1 inhibition. Gut.

[24] Enzo E, Santinon G, Pocaterra A, Aragona M, Bresolin S, Forcato M (2015). Aerobic glycolysis tunes YAP/TAZ transcriptional activity. Embo j.

[25] TeSlaa T, Ralser M, Fan J, Rabinowitz JD (2023). The pentose phosphate pathway in health and disease. Nat Metab.

[26] Patra KC, Hay N (2014). The pentose phosphate pathway and cancer. Trends Biochem Sci.

[27] Liu H, Xu L, Yue S, Su H, Chen X, Liu Q (2024). Targeting N4-acetylcytidine suppresses hepatocellular carcinoma progression by repressing eEF2-mediated HMGB2 mRNA translation. Cancer Commun (Lond).

[28] Shah SS, Stone EF, Francis RO, Karafin MS (2024). The global role of G6PD in infection and immunity. Front Immunol.

[29] Zhang Y, Solinas A, Cairo S, Evert M, Chen X, Calvisi DF (2021). Molecular Mechanisms of Hepatoblastoma. Semin Liver Dis.

[30] Zheng J, Tan Y, Liu X, Zhang C, Su K, Jiang Y (2022). NAT10 regulates mitotic cell fate by acetylating Eg5 to control bipolar spindle assembly and chromosome segregation. Cell Death Differ.

[31] Zhang Y, Xu L, Ren Z, Liu X, Song J, Zhang P (2022). LINC01615 maintains cell survival in adaptation to nutrient starvation through the pentose phosphate pathway and modulates chemosensitivity in colorectal cancer. Cell Mol Life Sci.

[32] Mele L, Paino F, Papaccio F, Regad T, Boocock D, Stiuso P (2018). A new inhibitor of glucose-6-phosphate dehydrogenase blocks pentose phosphate pathway and suppresses malignant proliferation and metastasis *in vivo*. Cell Death Dis.

[33] Zhang Y, Lei Y, Dong Y, Chen S, Sun S, Zhou F (2024). Emerging roles of RNA ac4C modification and NAT10 in mammalian development and human diseases. Pharmacol Ther.

[34] Zeng T, Li B, Shu X, Pang J, Wang H, Cai X (2023). Pan-cancer analysis reveals that G6PD is a prognostic biomarker and therapeutic target for a variety of cancers. Front Oncol.

[35] Deng P, Li K, Gu F, Zhang T, Zhao W, Sun M (2021). LINC00242/miR-1-3p/G6PD axis regulates Warburg effect and affects gastric cancer proliferation and apoptosis. Mol Med.

[36] Chen X, Xu Z, Zhu Z, Chen A, Fu G, Wang Y (2018). Modulation of G6PD affects bladder cancer via ROS accumulation and the AKT pathway *in vitro*. Int J Oncol.

[37] Jacquier V, Gitenay D, Cavaillès V, Teyssier C (2022). The Transcription Coregulator RIP140 Inhibits Cancer Cell Proliferation by Targeting the Pentose Phosphate Pathway. Int J Mol Sci.

[38] Zheng X, Wang Q, Zhou Y, Zhang D, Geng Y, Hu W (2022). N-acetyltransferase 10 promotes colon cancer progression by inhibiting ferroptosis through N4-acetylation and stabilization of ferroptosis suppressor protein 1 (FSP1) mRNA. Cancer Commun (Lond).

[39] Hansen CG, Ng YL, Lam WL, Plouffe SW, Guan KL (2015). The Hippo pathway effectors YAP and TAZ promote cell growth by modulating amino acid signaling to mTORC1. Cell Res.

[40] Doxtater K, Tripathi MK, Sekhri R, Hafeez BB, Khan S, Zafar N (2023). MUC13 drives cancer aggressiveness and metastasis through the YAP1-dependent pathway. Life Sci Alliance.

[41] Zhu M, Peng R, Liang X, Lan Z, Tang M, Hou P (2021). P4HA2-induced prolyl hydroxylation suppresses YAP1-mediated prostate cancer cell migration, invasion, and metastasis. Oncogene.

[42] Koo JH, Guan KL (2018). Interplay between YAP/TAZ and Metabolism. Cell Metab.

[43] Du K, Hyun J, Premont RT, Choi SS, Michelotti GA, Swiderska-Syn M (2018). Hedgehog-YAP Signaling Pathway Regulates Glutaminolysis to Control Activation of Hepatic Stellate Cells. Gastroenterology.

