# Supplementary material for: YAP-activated NAT10 promotes hepatoblastoma progression by activating the pentose phosphate pathway
Source: Int J Biol Sci. 2025 Apr 13;21(6):2864–79. doi: 10.7150/ijbs.109552 (PMC12035897; doi:10.7150/ijbs.109552)
Supplement: Supplementary file 1 — Supplementary figures. [file ijbsv21p2864s1.pdf]

Figure legends

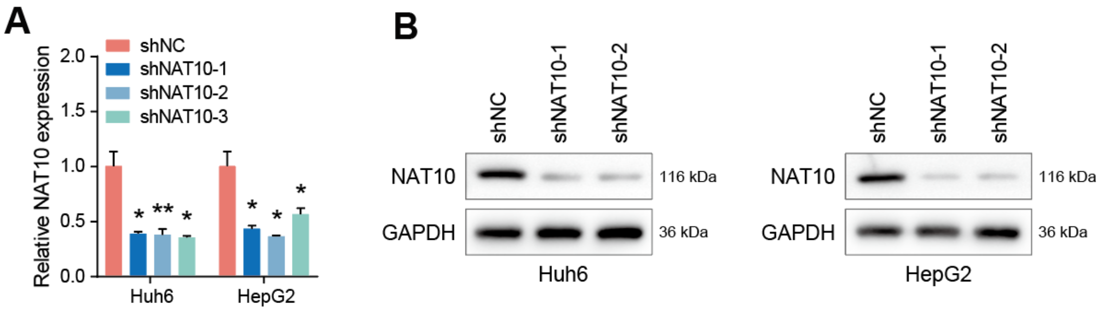

Fig S1. NAT10 expression is upregulated in hepatoblastoma, promoting HB cell proliferation and metastasis. (A) qRT-PCR detected the expression of NAT10 with knockdown in HB cell lines. (B) Western blot detected the protein expression of NAT10 with knockdown in HB cell lines.

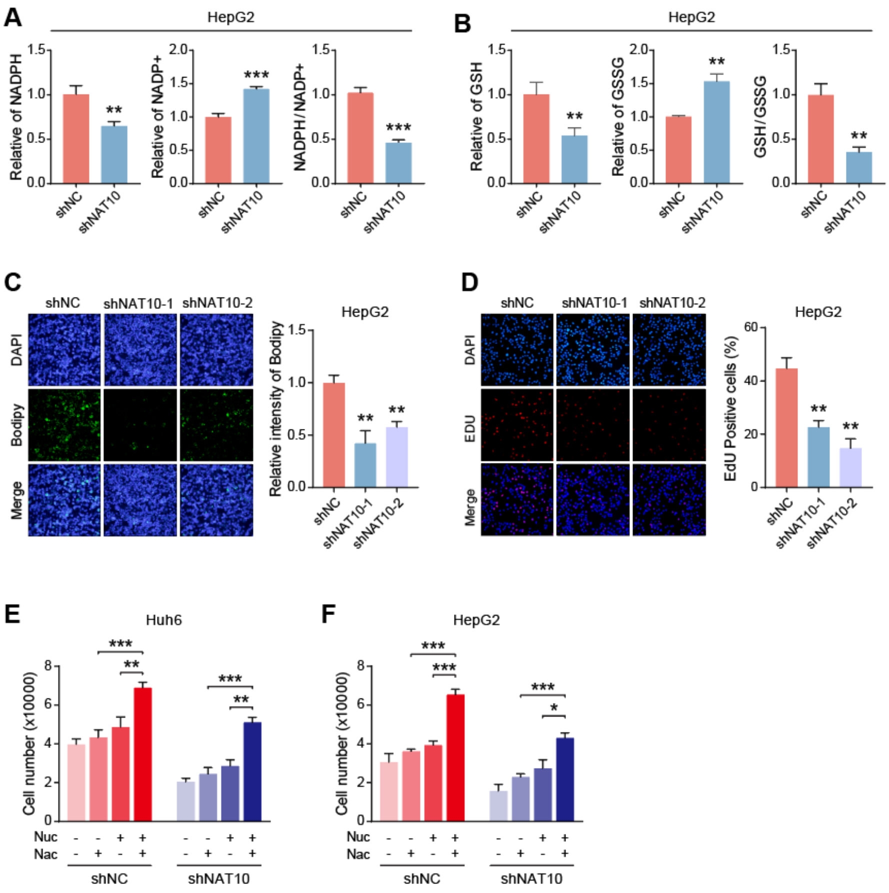

Fig S2. NAT10 upregulates the pentose phosphate pathway (PPP) in HB cells. (A) Kit detection of NADPH level and production capacity in NAT10-deficient HB cells. (B) Kit detection of GSH production level in NAT10-deficient HB cells. (C) Bodipy detection of lipid synthesis level in NAT10-deficient HB cells. (D) EDU assay for measuring the proliferation capacity of NAT10-deficient HB cells. (E-F) CCK-8 assay for assessing cell viability level after addition of Nuc and Nac.

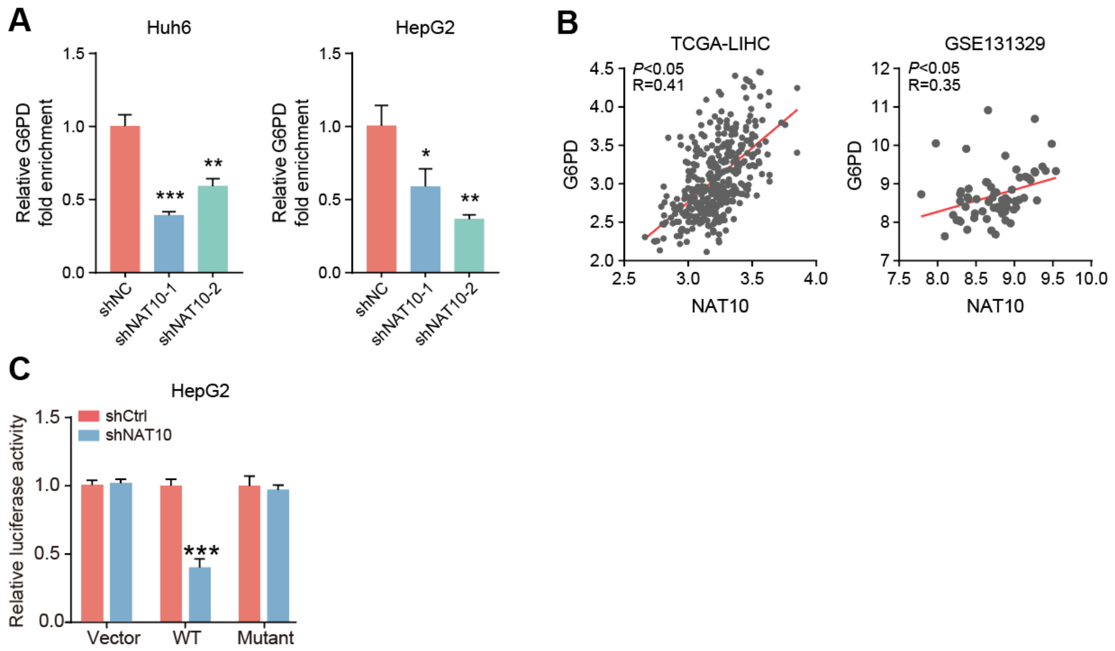

Fig S3. NAT10 mediates ac4C modification to upregulate G6PD expression. (A) acRIP-qRT-PCR detection of G6PD ac4C modification level after NAT10 knockdown. (B) Correlation analysis of NAT10 and G6PD expression in the TCGA database and tissue samples. (C) Dual-luciferase assay detected fluorescence intensity after NAT10 knockdown in HepG2 cell.

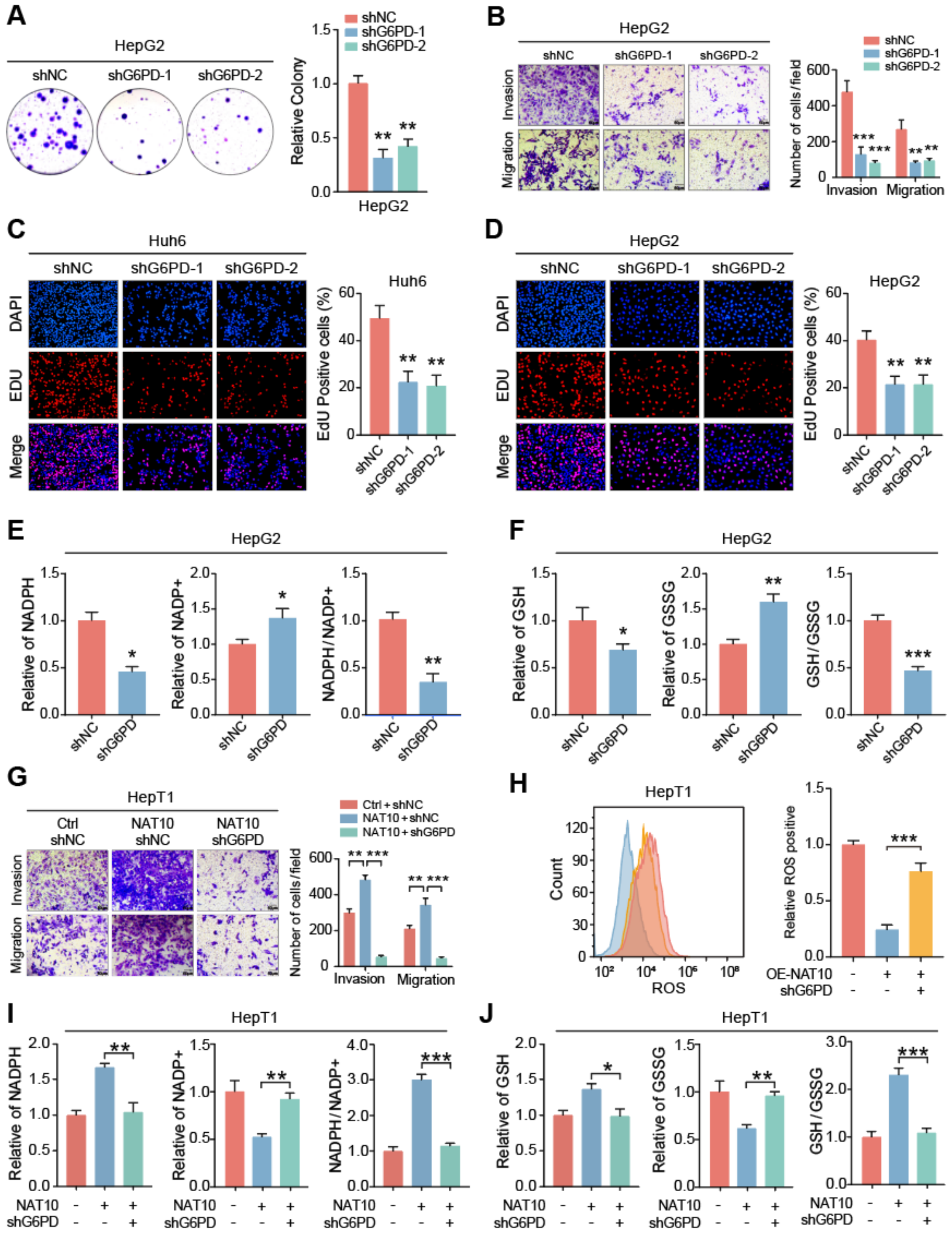

Fig S4. NAT10 promotes malignant progression of HB by upregulating the G6PD-dependent PPP pathway. (A-D) Cell proliferation and invasion experiments assessing the proliferation and migration capabilities of G6PD-deficient HB cells. (E-F) Kits detected NADPH content and generation and GSH generation levels in G6PD-deficient HB cells. (G) The Transwell assay showed that knocking down G6PD could inhibit the increase in migration ability induced by NAT10 overexpression.(H-J) Detection of ROS levels, NADPH content and generation levels, and GSH generation levels in HepT1 cells using kits.

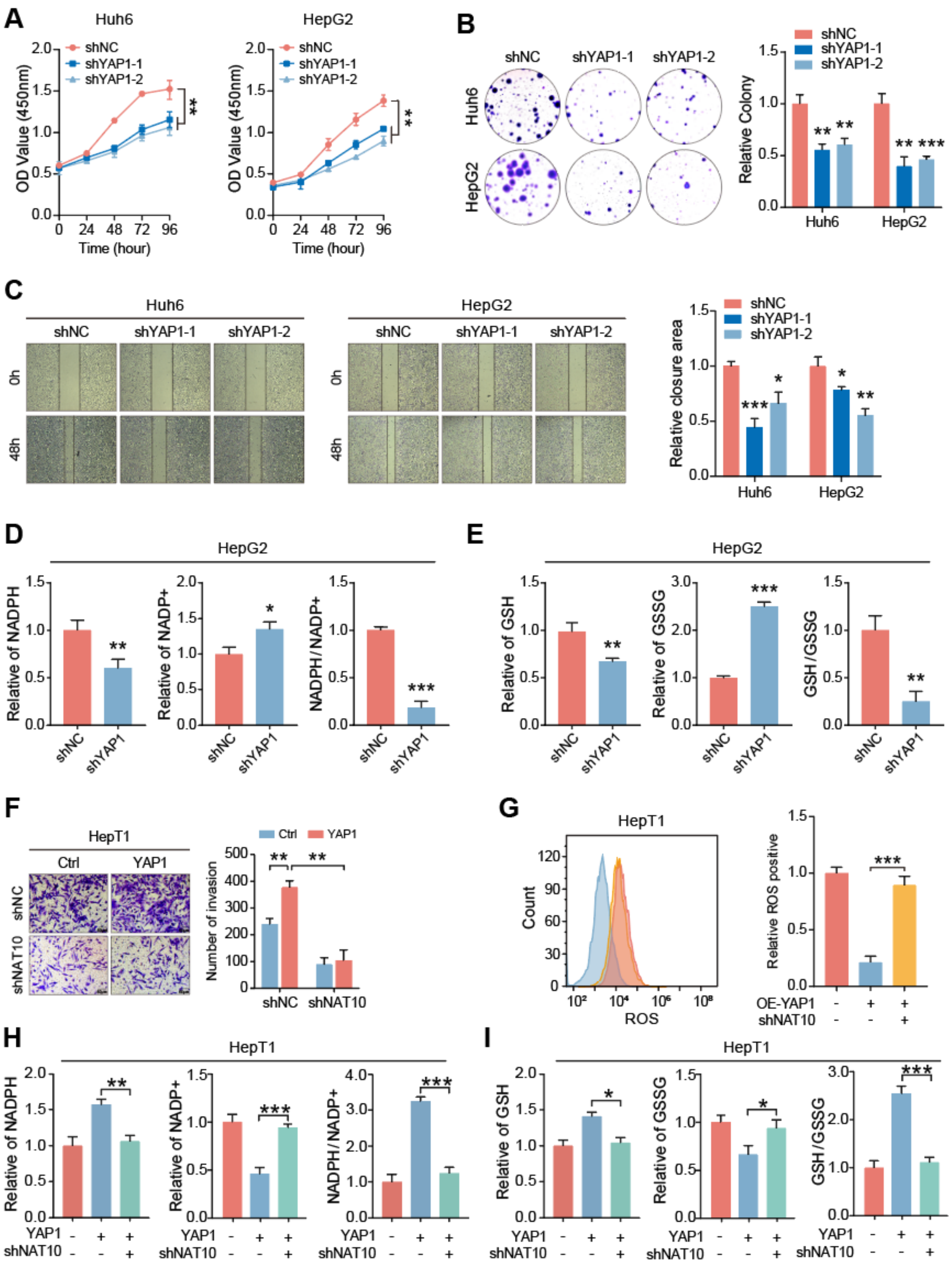

Fig S5. YAP1 regulates NAT10 expression and activates PPP, thereby promoting malignant progression of HB. (A-B) Cell proliferation assays detected the proliferative capacity of YAP1-deficient HB cells. (C) The wound healing assay assessed the migration level of YAP1-deficient HB cells. (D-E) Kit assays assessed NADPH content and generation and GSH generation levels in YAP1-deficient HB cells.(F) Transwell assays showed that NAT10 knockdown inhibited the enhancement of invasion induced by YAP1 overexpression.(G-I) Kit assays detected ROS levels, NADPH content and generation levels, and GSH generation levels in HepT1 cells.

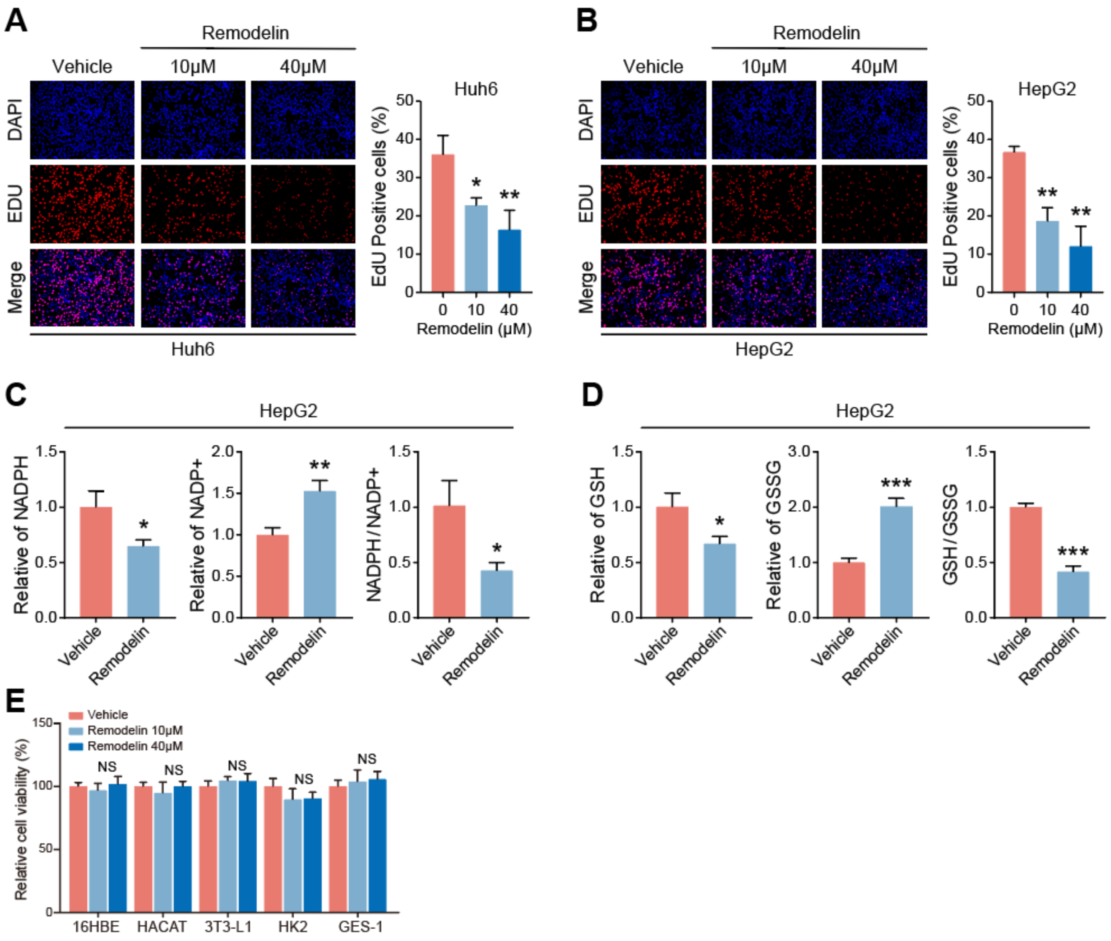

Fig S6. The NAT10 inhibitor Remodelin effectively inhibits the malignant progression of HB. (A-B) The EDU assay showed that Remodelin inhibited proliferation capacity. (C-D) Kit assays detected NADPH content and generation and GSH generation levels in HB cells treated with Remodelin. （E）Effect of Remodelin on cell viability measured by MTT assay.
